# A Natural Low Phytic Acid Finger Millet Accession Significantly Improves Iron Bioavailability in Indian Women

**DOI:** 10.3389/fnut.2021.791392

**Published:** 2022-03-24

**Authors:** Bellam H. Rajashekar Reddy, Prashanth Thankachan, Masoami Hatakayama, Netravati Hiremath, Diego Moretti, Yellodu A. Nanjareddy, Mathi B. Thumilan, Ramapura L. Ravikumar, Shamprasad Phadnis, Beena Bose, Lucy Poveda, Geetha Kalaiah, Michael B. Zimmermann, Kentaro K. Shimizu, Ralph Schlapbach, Anura V. Kurpad, Sheshshayee M. Sreeman

**Affiliations:** ^1^Division of Nutrition, St. John's Research Institute, St. John's National Academy of Health Sciences, Bengaluru, India; ^2^Department of Evolutionary Biology and Environmental Studies, University of Zurich, Zurich, Switzerland; ^3^Functional Genomics Center Zurich, Zurich, Switzerland; ^4^All India Coordinated Research Project (Foods and Nutrition), University of Agricultural Sciences, Bengaluru, India; ^5^Laboratory for Human Nutrition, Institute of Food, Nutrition and Health, ETH Zurich, Zurich, Switzerland; ^6^Department of Crop Physiology, University of Agricultural Sciences, Bengaluru, India; ^7^Department of Biotechnology, University of Agricultural Sciences, Bengaluru, India; ^8^Department of Physiology, St. John's Medical College, St. John's National Academy of Health Sciences, Bengaluru, India

**Keywords:** finger millet, grain phytic acid, iron deficiency anemia (IDA), bioavailability, stable isotope

## Abstract

Iron deficiency and anemia are common in low- and middle-income countries. This is due to a poor dietary iron density and low iron absorption resulting from the high inhibitory phytic acid content in cereal and millet-based diets. Here, we report that a naturally occurring low phytic acid finger millet accession (571 mg 100 g^−1^), stable across three growing seasons with normal iron content (3.6 mg 100 g^−1^), increases iron absorption by 3-folds in normal Indian women. The accessions differing in grain phytic acid content, GE 2358 (low), and GE1004 (high) were selected from a core collection of 623 accessions. Whole genome re-sequencing of the accessions revealed significant single nucleotide variations segregating them into distinct clades. A non-synonymous mutation in the *EcABCC* phytic acid transporter gene between high and low accessions could affect gene function and result in phytic acid differences. The highly sensitive dual stable-isotope erythrocyte incorporation method was adopted to assess the fractional iron absorption. The low phytic acid accession resulted in a significantly higher iron absorption compared with the high phytic acid accession (3.7 vs. 1.3%, *p* < 0.05). The low phytic acid accession could be effective in preventing iron deficiency in regions where finger millet is habitually eaten. With its low water requirement, finger millet leaves low environmental footprints and hence would be an excellent sustainable strategy to mitigate iron deficiency.

## Introduction

The prevalence of iron deficiency (ID) and iron deficiency anemia (IDA) is high in many parts of the world (1). For example, in India, the prevalence of all-cause anemia in women of reproductive age (WRA) was reported to be 53% in a national survey (2). Globally, the most significant contributor to anemia is thought to be the deficiency of iron (3). In India, where the daily diet has a low iron density of about 8.5 mg 1,000 kcal^−1^ (4), a mild risk of ID could occur with cereal based diets, which may not deliver the complete daily iron requirement of 15 mg day^−1^ in WRA (5) and 18 mg day^−1^ in adolescent children (6). Since the net iron absorbed is the product of the iron content of food and its bioavailability, either of these (or both) could be altered when trying to improve the iron availability to humans. Thus, one strategy is to increase the iron density in staple food crops either by conventional breeding, biotechnology techniques, or agronomic approaches (7). However, this does not address iron absorption and could even lower it. For example, in common beans (8) and pearl millet (9), the net iron absorbed from iron biofortified varieties was higher compared to control varieties. However, the fractional iron absorption remained the same (in biofortified pearl millet) but was lower (in biofortified common beans) compared to their respective controls. Other strategies to improve iron density, such as food iron fortification, are also likely to have a low impact since the absorption of iron from fortified staple foods (that are high in inhibitors) in efficacy trials has been uniformly low, ranging from about 1–2% (10–12). It is known that delivering high doses of relatively non-bioavailable iron to the intestinal mucosa can result in higher hepcidin levels that reduce iron absorption by up to 50% (13), and therefore the strategy of increasing iron density of grains, while more subtle in approach than supplementation, might still eventually be limited in success in the long term. There may also be a downside in the delivery of more iron into the body, whether it is absorbed from the intestine or not, since unabsorbed iron has a negative impact on the gut microbiome (14), while increased absorbed iron can increase the risk of many chronic diseases through a variety of effects (15).

The other, and perhaps safer and better, strategy in the prevention of ID is to improve iron absorption. The usual monotonous cereal-based and generally vegetarian diet are inhibitory due to the high content of iron absorption inhibitors such as phytic acid and polyphenols, and the low content of absorption enhancers like vitamin C. Phytic acid (*myo-*Inositol-1,2,3,4,5,6-hexa*kis*phosphate) a phosphorous storage molecule in plant seeds and ubiquitous to eukaryotic cells (16). During seed development, phytic acid is accumulated in the seed and readily chelates mineral cations like Mg, Fe, K, Cu, and Zn to form mixed salts known as phytate or phytin, which act as an antioxidant in the seed (17, 18). From the perspective of the plant, these mixed salts of phytic acid act as a store for phosphorous, inositol, and mineral cations (19). These stored nutrients are broken down and retrieved during the seed germination process by the enzymatic action of phytase (20). Monogastric animals, including humans, lack phytase in their gastrointestinal tract, and this makes the phytate bound nutrients unavailable for absorption (21). One way to overcome these inhibitory effects is by increasing diet diversity that includes increased intake of fruits that contain vitamin C, which is a facilitator of iron absorption. Vitamin C overcomes the inhibitory effect of phytate or polyphenols on iron absorption when available in an appropriate molar ratio to the meal iron content (4, 22). Another key strategy for increasing iron absorption from diets is by reducing the dietary phytic acid content (23), through the introduction of the phytase enzyme into grain flours (24), but requires specific conditions for effective enzymatic activity (25). This strategy will be effective only if most phytate is removed. For instance, in one study, 95% dephytinization of test meals made with biofortified beans resulted in an increase in fractional iron absorption by 51% compared to control (8). Though these methods are effective, they are expensive and possibly unsustainable. It might appear that the more sensible and sustainable option is to improve iron absorption by finding or developing low phytic acid cereal or millet grains for farming. Strategies to reduce the grain phytic acid (GPA) content of grain crops include either the development of low GPA mutants by altering the biosynthetic pathway and transport genes (26), or the identification and selection of natural variants with a low GPA content (27, 28).

This study evaluated how iron absorption could be improved in finger millet, which is culturally popular and habitually consumed in India (29) as well as in other parts of the world (30). It is rich in minerals like iron and calcium and has a high content of dietary fiber (31). However, although its iron content is thought to be high, its bioavailability is lower than other cereals (4.6% in ragi, 8.3% in white rice, and 11.2% in whole wheat *Atta* flour measured by isotopic meal-based iron absorption studies in women with ID) (32), due to its phytic acid and tannin content. Therefore, this study aimed to select a natural and consistently low GPA content finger millet accession from a collection of 623 accessions that represented their global diversity (33). Along with the phenotypic characteristics, allelic variations in the GPA biosynthetic pathway and transporter gene(s), from whole genome sequences of this accession, were compared with those of a selected high GPA accession. Finally, the translational potential of the low GPA content accession in promoting iron absorption was measured in human subjects using stable isotopes of iron (34).

## Materials and Methods

### Development of a Diversity Panel From a Core Collection of 623 Finger Millet Accessions

A “core” collection consisting of 623 finger millet accessions representing global diversity (33) was acquired from the All India Coordinated Research Project on small millets, GKVK campus, Bangalore, India. These were grown in a field experiment during the first production season of 2015, at the University of Agricultural Sciences (UASB), Bangalore (13.05°N, 77.34°E). Phenotyping for 18 quantitative traits was carried out and trait values were recorded following guidelines defined by International Board for Plant Genetic Resources (IBPGR, 1985) (35). The detailed crop production activities and the measurement of phenotypic traits are described in Supplementary Text A. For GPA measurement, bulk seed samples of each accession were dried in a hot air oven at 60°C for 48 h and ground to a fine flour by using a ball mill and stored in an airtight container until analysis. The GPA content in each of the core 623 finger millet accessions was measured in triplicates using a modified high throughput Wade colorimetric assay (36). A common finger millet accession (MR-6) was used to monitor inter assay variation and GPA values were accepted when the CV was <5% between the assays.

Molecular diversity analysis of the 623 core accessions was performed by using previously reported 35 simple sequence repeat (SSR) markers (37) (Supplementary Table 1) from finger millet. Protocol for genomic DNA isolation, specific reaction conditions to perform polymerase reactions, and marker scoring are described in Supplementary Text B.

The phenotypic and molecular data of the 623 accessions (2015) was analyzed using POWERCORE 1.0 Software (38) to develop a “diversity panel” of 350 finger millet accessions, representing the wide diversity present in the original core panel. The accessions from the diversity panel were grown again in production season 2016 at UASB as described earlier. In the 350 accessions grown, 75 showed poor germination and low crop stand. Therefore, these accessions were not considered for GPA analysis. To ascertain if the remaining 275 accessions were representative of 350 “diversity panel” accessions, the means, and variances of all 19 traits (including GPA) between 350 and 275 accessions were comparable.

### Selection of Finger Millet Accessions With High and Low GPA Content From the Diversity Panel

A two-step approach was undertaken for the final selection of low and high GPA content accessions for human testing. First, 19 accessions were selected in a stratified manner which is evenly distributed for GPA across the 275 diversity panel. Second, the GPA content was rigorously tested through a validating measurement at an external laboratory (Human Nutritional Laboratory, ETH, Zurich). This validating analysis used the modified Makower method (39) with certified wheat bran as quality control (phytic acid concentration range: 4.16–5.42 g 100 g^−1^). The iron content of the 19 selected accessions was also quantified at the Human Nutritional Laboratory, ETH, Zurich, using an Atomic Absorption spectrophotometer (AAS) (39).

Finally, from the selected 19 accessions, one high (GE 1004) and one low type (GE 2358), which showed consistently high and low GPA content in both production seasons, as well as consistent GPA values between the analytical laboratories, were selected for evaluation of iron bioavailability in human feeding trials. The selected two finger millet accessions were grown during the production season 2017 at UASB on a larger area of land (between July and November) to produce the quantity of seed required (~5 kg each) for the human iron bioavailability study.

### Identification of Allelic Variations in GPA Biosynthetic Pathway and Transport Genes

The whole genomes of the selected 19 accessions, were re-sequenced on the Illumina HiSeq4000 platform (Illumina, Inc., California, USA) by 150 bp paired-end libraries with an average 10X coverage of the genome. The sequenced reads were aligned to the updated PR202 reference sequence (Supplementary Text C) by BWA (v0.7.17) with trimming and filtering low quality reads by Trimmomatic (v 0.36) pre-processing. The aligned reads were analyzed and classified into A and B sub-genomes by EAGLERC (v1.1.1) software (40). For each of the 19 accessions, SNP calling was performed independently for the two sub-genomes by GATK (v4.1.2.0) which revealed 7,732,239 and 8,743,397 SNPs, respectively. False positive SNPs were removed by aligning the sequences of selected 19 accessions with the reference sequence of PR 202. Common SNPs in 19 accessions were considered false and omitted from further analysis.

To identify SNPs associated with GPA content, 18 genes involved in GPA biosynthetic pathway and transport (26) were selected from the rice genome database, IRGSP-1. BLAST comparison of these 18 genes with the draft genome sequence of PR202 was performed. Homologous sequences of these 18 genes among the 19 accessions were aligned to identify SNPs using GATK (v4.1.2.0). To assess the genetic diversity/relatedness across 19 accessions, the SNPs identified were subjected to Neighbor joining (NJ) tree and Principal component analyses (PCA) using VCF-Kit (ver. 0.2.9) and PLINK (ver. 1.90beta6.21). *In silico* analysis and protein structure prediction were performed to validate the SNP's between high and low GPA accessions (Supplementary Text D).

### Measurement of Iron Absorption in Humans From Low and High GPA Content Finger Millet Accessions

From the 2017 season, seeds of the selected accessions (GE2358; GE1004) were ground to a fine flour using a custom-made Teflon coated grinder equipped with titanium blades (Cingularity, Bengaluru) to avoid any external iron contamination. The flour was stored at 4°C until the test meals were prepared. A culturally acceptable unleavened flat bread (*Ragi roti*) was made from flour and used as a test meal. The recipe was standardized in the metabolic kitchen of the Division of Nutrition at St. John's Research Institute, Bengaluru (Supplementary Text E). The nutrient composition of the test meal is presented in (Supplementary Table 2) and was within the macronutrient requirements for a breakfast meal that provided one-fourth of daily energy and protein requirement of healthy sedentary women (41) and the remaining nutritional requirements of subjects were met through their habitual dietary intake (as lunch, dinner, and one snack).

A total of 20 healthy young women were screened from the students and junior staff population of UAS, Bengaluru, for the study. This study site was chosen as the preparation of *Ragi roti* is a commonly consumed breakfast/lunch meal in this population. About 10 subjects with no reported chronic medical illnesses, with no infection (C-reactive protein, CRP <10 mg L^−1^), who were not pregnant or lactating, with normal iron status (Hb > 12 g dL^−1^, serum ferritin >15 μg L^−1^) were included in the study. Those who were taking vitamin or mineral supplementation within the last month prior to the study were excluded. The study protocol was approved by the Institutional Ethical Committee at St. John's Medical College, Bengaluru, India (IERB No:87/2015), registered at the Clinical Trials Registry of India as CTRI/2020/01/02267, and informed written consent was obtained from all subjects.

On the baseline collection day (Day 1), participants reported to the study location at 8 AM after an overnight fast. Their height and weight were measured using standardized procedures followed by a basal venous blood sample (6 mL) collection in an EDTA vacutainer (2 mL, Becton Dickenson, NJ, USA) and serum collection tube (4 mL, Becton Dickenson, NJ, USA). Whole blood (2 mL collected in a plain vacutainer) for analysis of the basal Fe isotopic enrichment in Hb, was stored at −80°C. Serum (1.5 mL) obtained after refrigerated centrifugation of whole blood at 3,500 rpm (Eppendorf, 5810 R, Germany) was stored at −20°C until analysis.

A crossover study design using each millet type (high and low GPA content) was followed for measuring iron absorption. The absorption is measured as a daily single meal protocol, that is, the iron absorbed from a single test meal. The test meals were administered each morning for 6 consecutive days, and subjects randomly received either high or low GPA test meals for three days each in succession. Just prior to intake by the subject, the test meals containing low or high GPA content finger millet were extrinsically labeled with ^57^FeSO_4_ or ^58^FeSO_4_, respectively. The dose of ^57^FeSO_4_ and ^58^FeSO_4_ were prepared as described elsewhere (22). The isotopic label added to the meal was equivalent to ~33% of the meal's native iron content. The dose of isotope label administered was gravimetrically determined and evenly dispensed onto the test meal, just before administration. The subjects were instructed to refrain from food and drink consumption until 3 h after a meal, post which they continued their habitual dietary intake. For the remaining 5 days, the same test meal administration protocol was followed, except that on days 4–6, the alternate GPA content test meal was used, with the alternate Fe isotope. On Day 20, that is, 14 days after the last test meal administration, a fasted venous blood sample (6 mL) was collected from the subjects, aliquoted, and stored as described earlier. The concentrations of Hb, serum ferritin, and CRP were measured on day 1 and day 20 (Supplementary Text F). All measurements were performed in duplicate.

The shift in isotopic ratios of ^57^Fe/^56^Fe and ^58^Fe/^56^Fe in Hb of the blood samples was analyzed in duplicate as described by Walczyk et al. (42). After mineralization, chromatographic separation, and extraction of iron from blood samples, the iron isotopic composition of the samples was determined by NTIMS (Triton, Thermo, Bremen, Germany) with a multicollector system (Supplementary Text G). The amount of circulating isotope label was calculated on the basis of the shift in the isotopic ratios and the amount of circulating iron in the blood. Calculations were based on principles of dilution, and the non-monoisotopic nature of the labels was taken into consideration (42). Circulating iron was calculated on the basis of blood volume and Hb concentration and 80% incorporation of the absorbed iron into erythrocytes was assumed (34). The observed shift in iron isotope ratios was converted to fractional iron absorption using standard algorithms (42).

## Statistical Analysis

The data are presented as mean ±SD. The comparison of the 18 traits between the core collection (*n* = 623), diversity panel (*n* = 350), and the successfully grown set (*n* = 275) was performed by analysis of variance (ANOVA) followed by *post-hoc* Newman–Keuls tests. To assess the level of diversity captured in the diversity panel from the core collection, mean difference (MD%), coincidence rate (CR%), and variable rate (VR%) were calculated as described elsewhere (43). Shannon and Weaver's (44) diversity index (H‘) were used to measure and compare the phenotypic diversity for each trait in the core collection and diversity panel. Data for GPA content for both seasons was tested for normality using the Shapiro-Wilk test. Genetic, phenotypic, and environmental variances and their CV were estimated as described elsewhere (45, 46). GPA content was correlated between seasons and labs by Pearson correlation. The agreement between the analytical laboratory estimates of GPA content was evaluated by the Bland–Altman method (47). For the human iron absorption study, the number of subjects (*n* = 10) had 80% power to detect a significant difference of 50% in iron absorption between the two accession test meals. The paired student's *t*-test was used to test differences between the log transformed iron absorption values from the low and high GPA content accessions and reconverted for reporting. Statistical analyses were conducted using SPSS software (version 17.0) and differences were considered significant at *p* < 0.05.

## Results

### Development of Finger Millet Diversity Panel

The mean, range, and variance of the 19 traits of the 623 accessions of the core collection are given in Table 1. Out of the 35-simple sequence repeat (SSR) markers screened for molecular characterization of the core collection, 8 primer pairs showed polymorphism. Both phenotypic and molecular diversities were analyzed by POWERCORE, from which the diversity panel of 350 accessions was assembled representing the original diversity of the core collection. Of the 19 traits, the mean values of 14 traits were not significantly different (*p* > 0.05) between the diversity panel and the core collection. The mean difference (MD%) of traits, between the core collection and diversity panel, were within the acceptable range of <20% and the coincidence rate (CR%) of the latter was 99.3 ± 0.5 for all traits (Supplementary Table 3). The mean Shannon weaver diversity index (H') for the 19 traits in the core collection (6.34 ± 0.09) and diversity panel (5.75 ± 0.10) was comparable (Supplementary Table 3). When analyzed for their GPA content (Supplementary File 1), the core collection showed significant variability between accessions (ANOVA, *p* < 0.001). The mean GPA content of the core collection (production season 2015) was 738 ± 92 mg 100 g^−1^, with the lowest content being 489 mg 100 g^−1^ and the highest being 951 mg 100 g^−1^ (Table 2).

**Table 1:** Comparison of mean, range, and variance for 19 quantitative traits in finger millet core collection (*n* = 623) and diversity panel (*n* = 350).

**SI No**.	**Trait**	**Range**		**Mean**			**Variance**	
		***n* = 623**	***n* = 350**	***n* = 623**	***n* = 350**	***p*-value**	***n* = 623**	***n* = 350**
1	Ear head emergence (days)	45.0–79.0	45.0–79.0	59.9	60.3	0.27	39.9	40.2
2	Plant height (cm)	45.0–146.0	45.0–146.0	94.4	95.5	0.34	278.6	329.2
3	Productive tiller (no plant^−1^)	1.4–8.4	1.4–8.4	3.9	4.0	0.15	1.3	1.6
4	Unproductive tiller (no plant^−1^)	0.1–2.0	0.1–2.0	0.5	0.5	0.12	0.1	0.1
5	Productive tiller ratio (%)	60.0–100.0	60.0–100.0	90.4	90.0	0.44	48.5	55.3
6	Leaf number (no plant^−1^)	11.9–96.0	12.2–96.0	36.7	38.4	0.05	156.6	194.5
7	Leaf area (cm^2^ plant^−1^)	268.2–4685.1	277.1–4685.1	1256.4	1349.0	0.03^*^	297747.1	406625.8
8	Specific leaf weight (mg cm^−2^)	0.5–10.0	0.5–10.0	6.0	6.0	0.49	1.0	1.3
9	Leaf dry weight (g plant^−1^)	1.7–22.4	1.8–22.4	7.4	8.0	0.02^*^	9.8	12.8
10	Leaf area index	0.9–15.6	0.9–15.6	4.2	4.5	0.03^*^	3.3	4.5
11	Stem dry weight (g plant^−1^)	6.9–104.7	6.9–104.7	32.1	34.2	0.04^*^	169.4	223.7
12	Mean ear head weight (g ear^−1^)	0.7–12.4	0.7–12.4	4.9	5.0	0.37	4.0	4.9
13	Ear head weight (g plant^−1^)	2.9–53.9	2.9–53.9	18.7	19.7	0.07	65.6	87.5
14	Seed yield (g plant^−1^)	2.0–43.4	2.0–43.4	13.7	14.4	0.11	38.3	51.2
15	Total dry matter (g plant^−1^)	11.5–181.1	11.5–181.1	58.6	62.5	0.03^*^	517.1	695.7
16	Threshing (%)	41.9–90.9	42.1–89.7	72.9	72.4	0.31	57.4	63.8
17	Test weight (g 1,000 seeds^−1^)	1.0–3.6	1.0–3.6	2.5	2.4	0.79	0.2	0.2
18	Harvest Index	0.05–0.45	0.05–0.45	0.2	0.2	0.59	0.0	0.0
19	Grain phytic acid (mg 100 g^−1^)	488.6–951.0	497.1–951.0	738.0	727.9	0.10	8463.5	8269.2

* *Significantly different at p < 0.05 based on ANOVA and post-hoc Newman-Keuls test*.

**Table 2:** Descriptive statistics for GPA content in finger millet accessions across generations.

**SI No**.	**Parameters**	**Year 2015**	**Year 2015**	**Year 2016**
		***n* = 623**	***n* = 275**	***n* = 275**
1	Mean (mg 100 g^−1^)	738	721	658
2	Minimum (mg 100 g^−1^)	489	497	478
3	Maximum (mg 100 g^−1^)	951	903	887
4	Standard deviation (SD)	92	90	80
5	Standard error mean (SEM)	9	9	12
6	Coefficient of variation (CV)	12.5	12.4	12.2
7	*p*-value (between accessions)^*^	<0.001	<0.001	<0.001

* *ANOVA was performed for GPA content and was significant at p < 0.05*.

**Figure 1:**
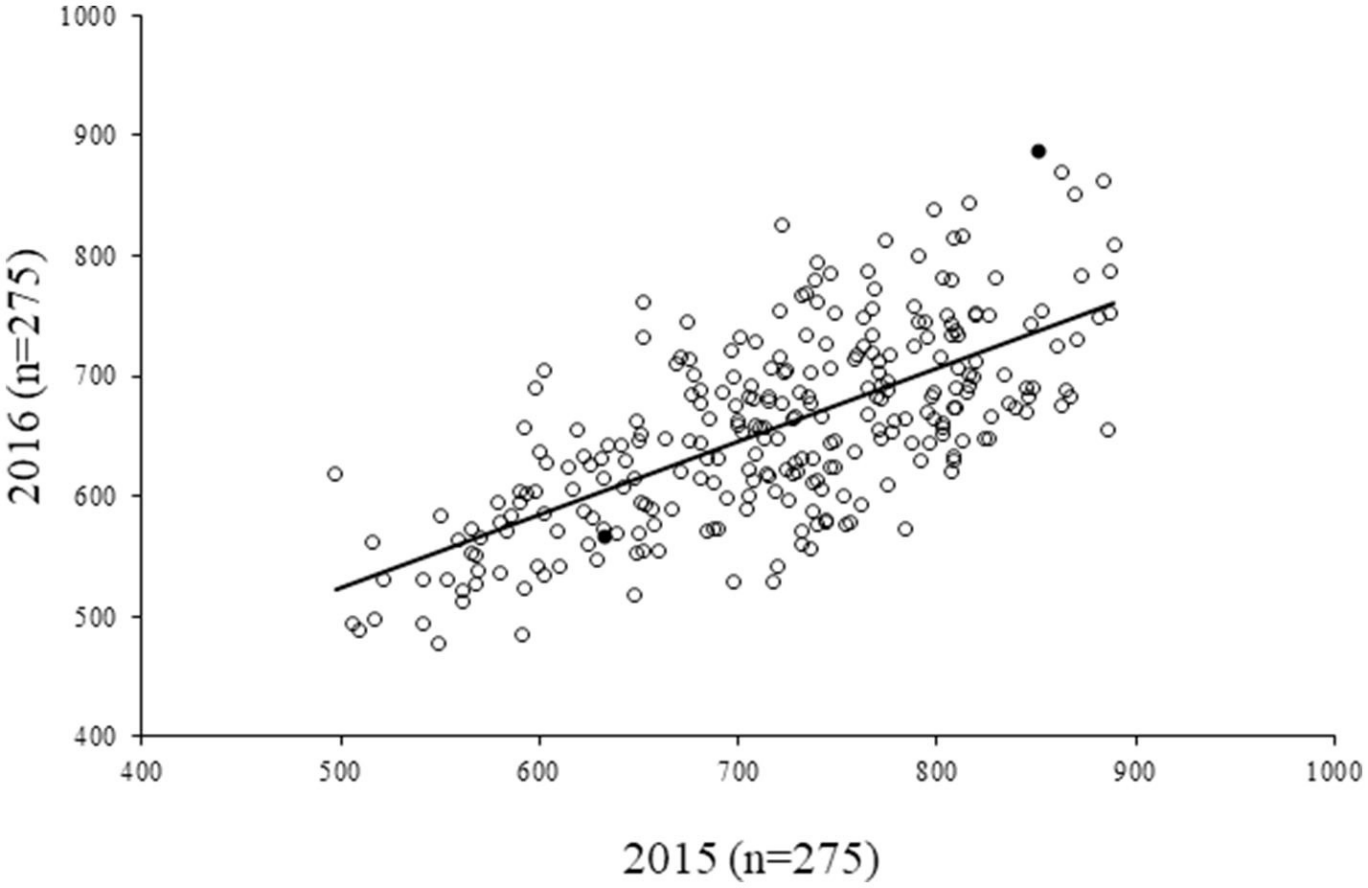
Correlation of GPA content in 275 finger millet accessions between two seasons (2015 and 2016). *r*^2^ = 0.472; *r* = 0.688, *p* < 0.001. Filled circles represent the accessions that were eventually selected for human iron absorption studies.

For the diversity panel, a subset of 275 accessions of the original selection (350 accessions) was successfully grown in the second production season 2016; these 275 accessions represented the diversity panel for a further selection of low and high GPA content accessions. There were no significant differences between the selected set of 350 and the successfully grown set of 275 accessions in all 19 traits studied (Supplementary Table 4). The GPA content of the 275 accessions is presented in Supplementary File 1. Analysis of variance for GPA content revealed significant variability between accessions (*p* < 0.001). The mean GPA content was 658 ± 80 mg 100 g^−1^ with the lowest content being 478 mg 100 g^−1^ and the highest being 887 mg 100 g^−1^ (Table 2). Analysis of genetic determinants of GPA showed values of phenotypic coefficient of variation of 12.68 and 12.40 and genotypic coefficient of variation of 12.50 and 11.98, respectively, for the two production seasons (Table 3). The estimates for heritability (h^2^) and genetic advance over mean for GPA content were 97.2 and 25.4, respectively, during production season 2015, and 93.4 and 24.0, respectively, for production season 2016 (Table 3). The GPA content among the 275 accessions was significantly and positively correlated between the two production seasons of 2015 and 2016 (*r* = 0.688, *p* < 0.001, Figure 1) indicating consistency of the trait. However, the mean GPA content differed significantly (*p* < 0.001) between the two production seasons.

**Table 3:** Estimates of genetic parameters for GPA content in finger millet core collection and diversity panel.

**SI No**	**Genetic parameters**	**Year 2015**	**Year 2015**	**Year 2016**
		***n* = 623**	***n* = 275**	***n* = 275**
1	GV	8493.1	7855.1	6199.5
2	PV	8738.6	8097.4	6637.7
3	EV	245.5	242.4	438.2
4	GCV	12.5	12.3	12.0
5	PCV	12.7	12.5	12.4
6	ECV	2.1	2.2	3.2
7	H^2^	97.2	97.0	93.4
8	GAM (%)	25.4	25.0	23.9

*GV, PV, and EV, Genetic phenotypic and environmental variances; GCV, PCV, and ECV, Genetic, phenotypic and environment coefficient of variation; H^2^, broad sense heritability; GAM, Genetic advance over mean*.

### Selection of High and Low GPA Finger Millet Accessions

In the final selection of a low GPA content accession (and a high GPA content accession as control) for the measurement of iron bioavailability in humans, 19 accessions were selected from the diversity panel, to represent its range of GPA content. In these 19 accessions, the GPA content was confirmed at an external laboratory using the Makower method of estimation. The GPA values from the Wade method at UAS Bengaluru, and the Makower method at ETH Zurich correlated significantly (*r*^2^ = 0.773, *p* < 0.001, Supplementary Figure 1). A Bland–Altman analysis of differences in the GPA content between the two methods showed a systematic difference of 68 mg 100 g^−1^ seed, while the random difference was ±100 mg 100 g^−1^ (Supplementary Figure 2). There was no significant correlation between the magnitude of the GPA content and the difference between the methods.

The GPA content of the selected 19 accessions from the diversity panel was examined for its stability during the two growing seasons of 2015 and 2016. From these selected 19 accessions, two contrasting GPA accessions which showed a consistently low (GE 2358) and high (GE 1004) GPA content were selected. The variation across the two seasons was <10% for the two selected accessions (Supplementary Table 5). The two selected accessions were grown again during the production season 2017 to produce adequate grains for human feeding trials to measure iron bioavailability. The stability of the GPA content of these selections between the three growing seasons was good and varied by a mere 36.4 mg 100 g^−1^ for GE 2358 and 67.1 mg 100 g^−1^ for GE 1004. In addition, the GPA content for these two accessions differed by <1% in the inter-laboratory analytical comparison. Thus, GPA content estimates for the final selection of low and high GPA accessions are reported as estimated by the Makower method. The low GPA accession, GE 2358, had 571 mg 100 g^−1^ while the high GPA accession, GE 1004, had 757 mg phytic acid in 100 g of grains. The iron content of these two accessions was 3.6 and 2.8 mg 100 g^−1^ in GE 2358 and GE 1004, respectively, and their phytate:iron molar ratios were 13.5:1 and 23.1:1, respectively. Thus, the low GPA accession also had a slightly higher iron content. In terms of grain yield during the 2016 production season, the low GPA content accession, GE 2358, had a slightly lower grain yield (by 9.9%) compared to the current Indian market variety (GPU 28).

### Allelic Variations in the GPA Biosynthesis and Transport Genes Between the High and Low GPA Finger Millet Accessions

**Figure 2:**
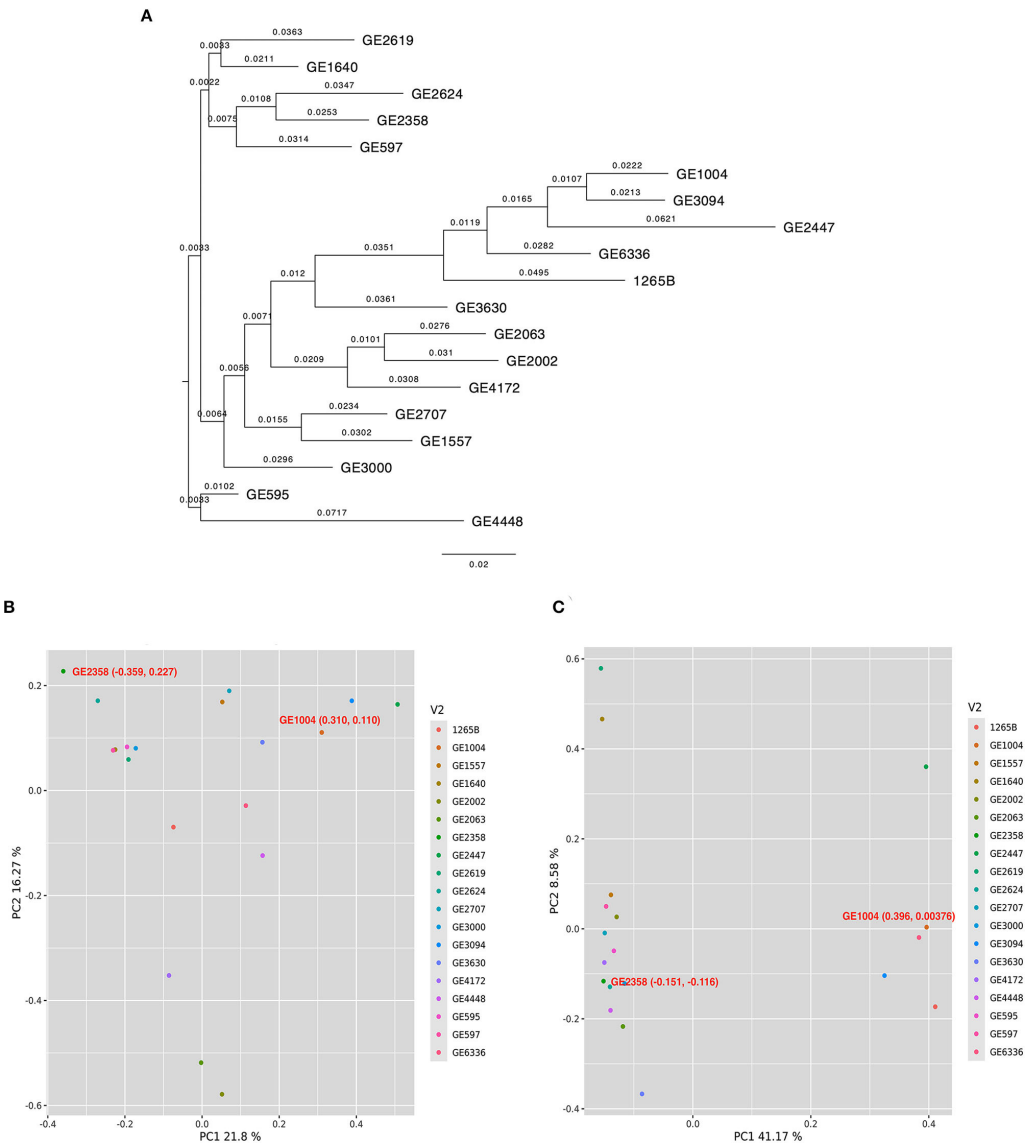
Diversity and principal component analysis for 19 finger millet accessions. **(A)** Neighbor joining tree to assess the genetic relatedness among 19 finger millet accessions. Principal component analysis for 19 finger millet accessions with identified SNPs across 66 matching sequences for A sub-genome **(B)** and B sub-genome **(C)**. Filled circles with different colors in scattered plots indicate the different accessions.

The 18 selected genes from the rice genome database (IRGPSV) when compared through a BLAST revealed 66 matching sequences across both A and B sub-genomes of finger millet. Of the 18 genes, 16 had at least two copies in either A or B sub-genomes (i.e., at least five copies in the whole genome), and five genes had five copies in the two sub-genomes of PR 202 (Supplementary Table 6). Annotation of the entire sequences of the 66 matches among 19 accessions revealed an average of 25.7 and 25.2 SNPs per accession in the entire gene sequence and an average of 0.78 and 0.76 SNPs per gene, on A and B sub-genomes, respectively. Further, we detected 123 and 186 SNPs on the coding regions (exons) among the selected 19 accessions. The average SNP in coding regions of each gene was 0.2 and 0.3, respectively, on the A and B sub-genomes. These genic SNPs segregated the 19 selected accessions into distinct clades as revealed by a Neighbor joining (NJ) tree. The low and high GPA accessions were separated with a significant genetic distance (Figure 2A). The mean GPA values of the accessions in the two clades differed significantly (*p* = *0.04*) in both production seasons (Supplementary Table 7). Principal component analysis of genic SNPs from A sub-genomes was scattered and did not separate contrast accessions (Figure 2B), while SNPs from B sub-genome distinctly separated two selected (GE 1004 and GE 2358) contrasting accessions (Figure 2C). The first two principal components explained 21.8% and 16.27% of the total variance respectively from the A sub-genome. GE 2358 (Low GPA) with coordinates −0.359, 0.227 and GE1004 (high GPA) with coordinates 0.310, 0.110 represented distinct differences. Similarly, the two principal components from the B sub-genome explained 41.17 and 8.58% of the total variance. GE 2358 with coordinates −0.151, −0.116, and GE 1004 with coordinates 0.396, 0.00376 again reiterated the genetic diversity between the selected accessions. From the analysis of both sub-genomes, GE 2358 was found to be governed by negative alleles while the high GPA accession, GE 1004, was governed by positive alleles, hence creating the differences in the observed GPA values.

**Figure 3:**
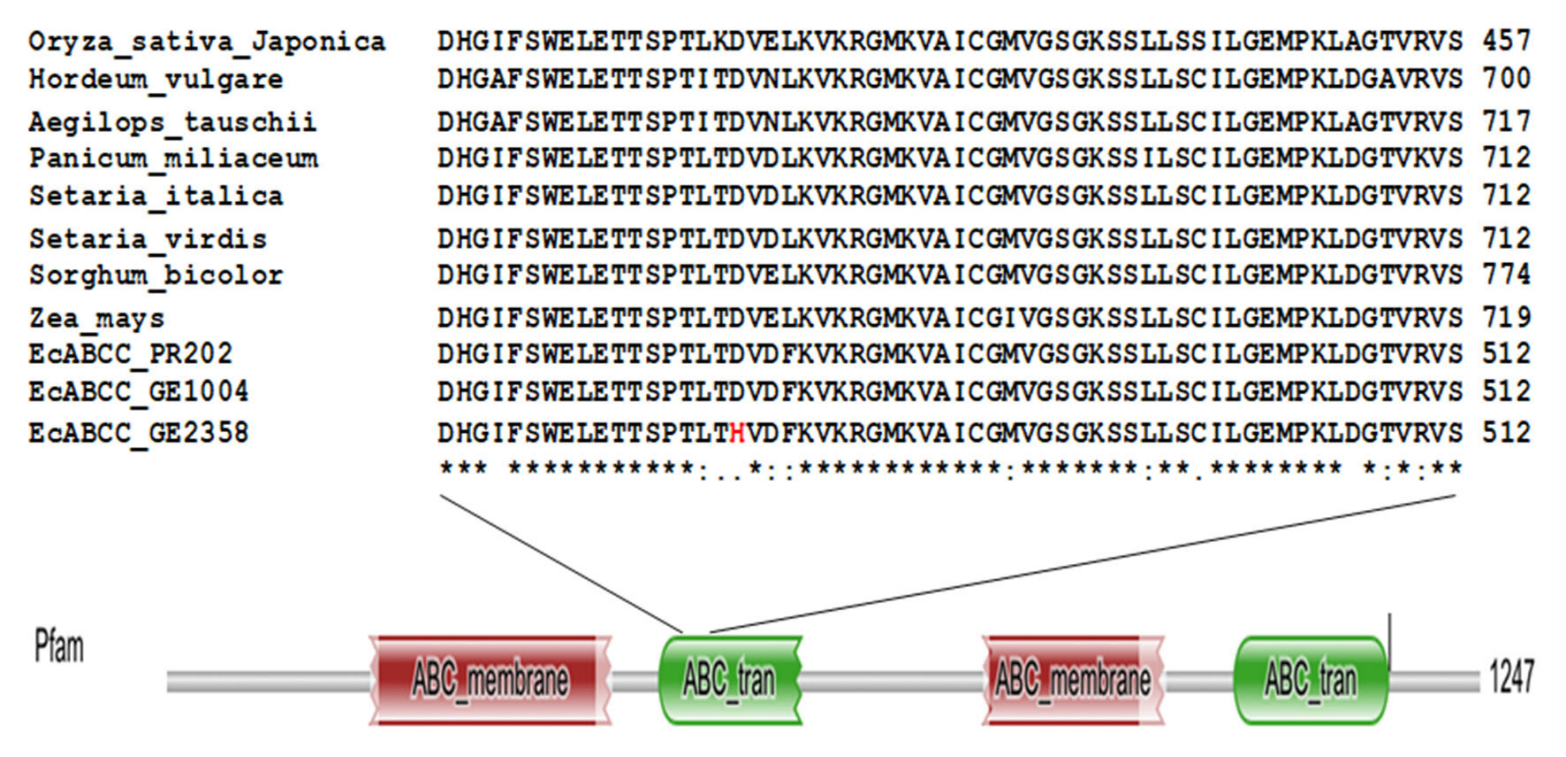
Multiple sequence alignment of deduced amino acid sequence of *EcABCC* from GE2358, GE1004, PR202, and monocot crop species and domain search analysis of *EcABCC*. The amino acid indicated in red color is replaced amino acid in low GPA accession.

Though there were several SNPs among the 19 accessions, we concentrated on SNPs in only the two contrasting accessions (Ge 1004 and GE 2358). Among all the SNPs one non-synonymous variant was found at the 1408th position of the nucleotide sequence of gb14539 the putative ATP-Binding cassette transporter-C family gene (phytate transporter gene *EcABCC*). The high GPA accession, GE 1004 had Guanine replaced with Cytosine in the low GPA accession GE 2358. This substitution changed the amino acid at the 470th position from aspartic acid in GE 1004, which is conserved among monocot crop species, to histidine in GE 2358 (Figure 3). A secondary structural analysis of the protein suggested that this mutation resulted in a change at the beta sheet region of the cytosol-facing ABC transporter domain (Supplementary Figure 3).

### Measurement of Iron Absorption From Low and High GPA Content Finger Millet Accessions

For the human iron absorption study, the recruited women's age was between 20 and 30 y, with a body mass index between 18.9 and 25.0 kg m^−2^. Anthropometric data and baseline iron status are presented in Table 4. All subjects had normal Hb levels (13.7 ± 0.8 g dL^−1^) with mean ± SD serum ferritin of 25.2 ± 10.1 μg L^−1^ and with no infection or inflammation (mean ± SD CRP: 1.6 ± 2.1 mg L^−1^). Because of the differing iron content of the two accessions, the iron content of the 3 test meals fed to the subjects for each accession, was slightly higher for the low GPA content GE 2358 (10.0 mg in total) than for the high GPA content GE 1004 (8.1 mg in total). The Fe isotopes (^57^Fe and ^58^Fe) were dispensed (1 mg of iron) onto the test meals (in total 3 mg of iron for 3 meals), thus constituting ~33% of the total iron in the meals. No subject reported any adverse reaction to the isotope feeding protocol during the study. The atom percent excess of the isotope labels of ^57^Fe and ^58^Fe at the end of the 14-day incorporation period in red blood cells was 0.27 and 1.02%, respectively.

**Table 4:** Baseline characteristics of the subjects.

**SI No**.	**Parameter**	**Mean ± SD (range)**
1	Age (y)	21.6 ± 3.0 (20–30)
2	Height (m)	1.6 ± 0.1 (1.4–1.6)
3	Weight (kg)	50.5 ± 4.2 (41.7–58.4)
4	BMI (kg M^−2^)	20.5 ± 1.5 (18.9–23.0)
5	Hemoglobin (g dL^−1^)	13.7 ± 0.8 (12.3–15.0)
6	Serum ferritin (μg L^−1^)	25.2 ± 10.1 (11.7–40.7)
7	CRP (mg L^−1^)	1.6 ± 2.1 (0.1–6.5)

*BMI, Body Mass Index; CRP, C-reactive protein*.

**Figure 4:**
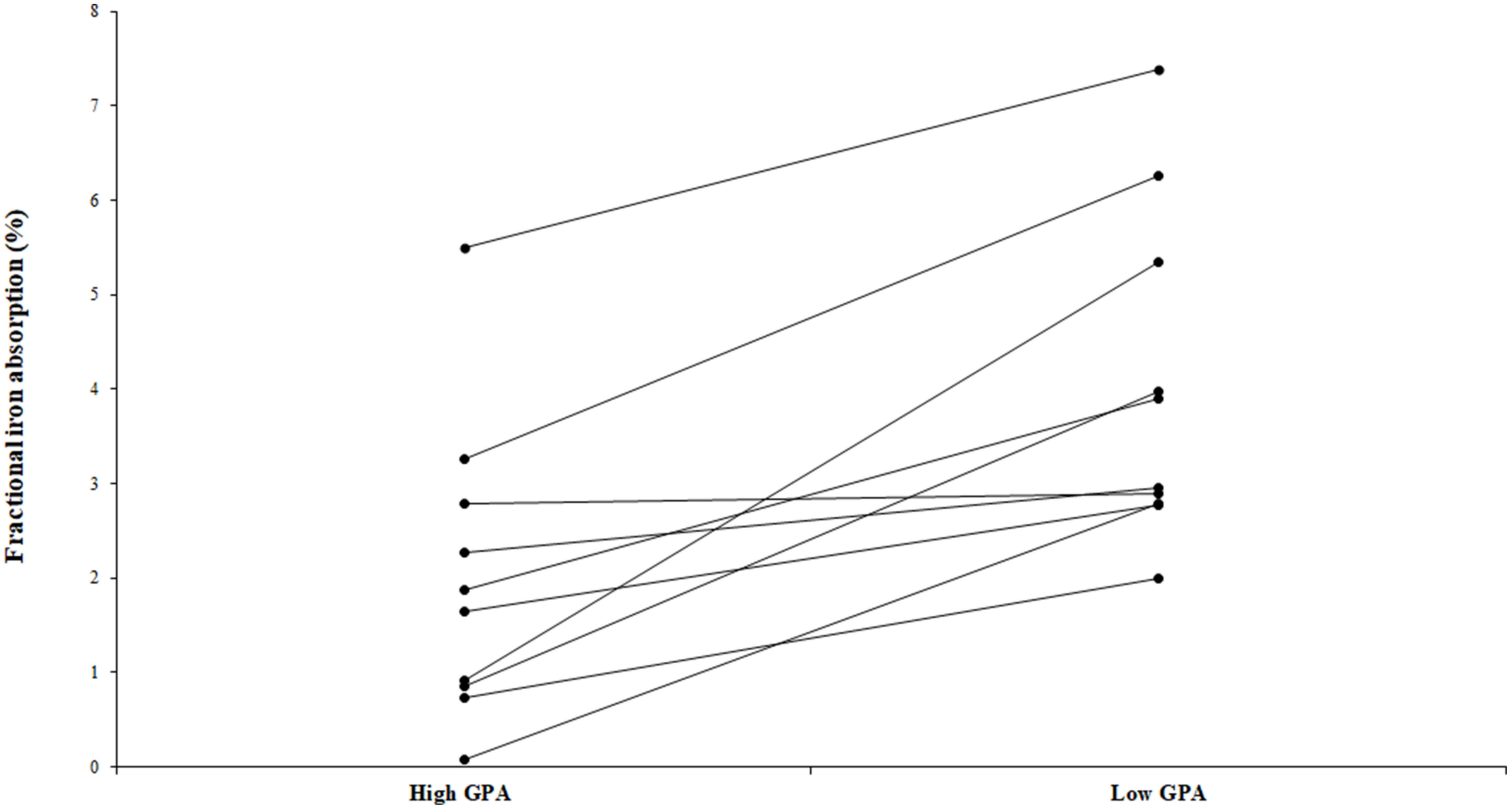
Fractional iron absorption from the low GPA and high GPA finger millet meals (*ragi roti, n* = 10). Both low and high GPA meals were administered to the same subject. Data points representing iron absorption of the same subject for high and low GPA test meals are connected with lines to illustrate the trend.

**Figure 5:**
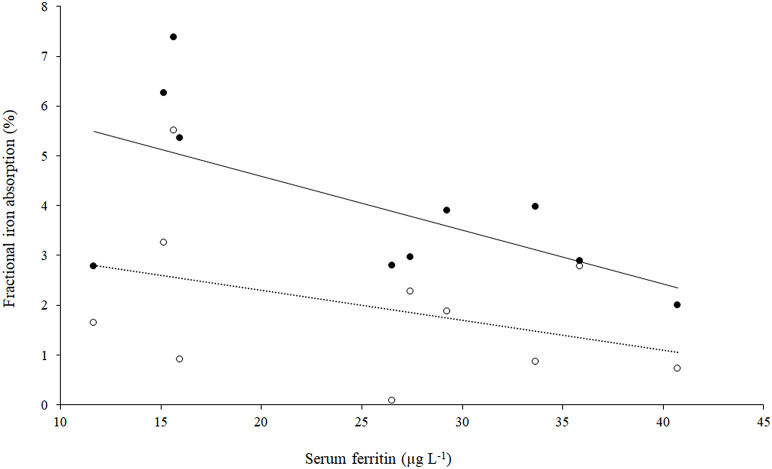
Scatter plot of iron absorption against serum ferritin in low GPA accession GE 2358 and high GPA accession GE 1004 (*n* = 10). Filled circles: Low GPA content finger millet accession; *r*^2^ = 0.38, *p* = 0.056, iron absorption = (−0.108 × serum ferritin) + 6.75. Open circles: High GPA content finger millet accession. *r*^2^ = 0.15, *p* = 0.275, iron absorption = (−0.060 × serum ferritin) + 3.50.

The fractional iron absorption from the low GPA test meal and high GPA test meal was significantly different (*p* < 0.05, Figure 4), at 3.7% (geometric mean, range: 2.0 to 7.4%) for the low GPA accession (GE 2358) in comparison to 1.3% (geometric mean, range: 0.1 to 5.5%) for the high GPA accession (GE 1004). Thus, the iron bioavailability was 2.9-fold higher on average with the low GPA accession meal intake. The low and high GPA test meals which contained 3.3 and 2.7 mg iron, contributed 0.122 mg (range: 0.066 to 0.243 mg) and 0.035 mg (range: 0.02–0.149 mg) respectively to daily physiological iron requirement (1.2 mg/d) after correcting for fractional iron absorption. For each accession, iron absorption had a negative but not significant correlation with serum ferritin for GE 2358 (*r*^2^ = 0.38, slope = −0.108 decrease in iron absorption per unit increase in serum ferritin, *p* = 0.056) and for GE 1004 (*r*^2^ = 0.15, slope = −0.060 decrease in iron absorption per unit increase in serum ferritin, *p* = 0.275). The negative slope indicates that subjects with a lower iron status (even within the normal range of serum ferritin) had a higher iron absorption (Figure 5).

## Discussion

Public health approaches to ID and IDA have involved the chemical iron fortification of foods, but unless there is adequate diet diversification, the absorption of fortified iron remains low (10), with low impact. There have also been global initiatives to biofortify crops, however, most of these have focused on increasing iron density (7, 48, 49), but have seen low to modest success (50, 51). A more effective strategy is to improve iron absorption from grains through the reduction of dietary inhibitors like phytic acid. This should be seen in conjunction with the revival and encouragement of the consumption of millets. With their relatively high iron, calcium, and fiber content (31), millet crops also have low input costs and water requirement (30) and have a sustainable, small ecological footprint. A further ecological benefit of reducing GPA content of the feed in non-ruminant (e.g., poultry) nutrition accrues from the reduced excretion of undigested phosphorus, with a possible impact of these reduced losses, through water run-off from farms, on the resultant eutrophication of water bodies (52).

In this study, a natural low GPA finger millet accession with adequate iron content, and stability of GPA content across growing seasons, was selected from a large germplasm collection with natural variation. This selection was hypothesized to increase native iron absorption. When measured in humans against a high GPA content finger millet contrast, using an accurate stable isotope method, showed an almost 3-fold increase in iron absorption. This increase in iron absorption is most likely due to the difference in phytic acid content (24%); while the iron content also was higher in the low GPA accession (by 24%), it is unlikely to increase iron absorption by this much. For example, an increase in the iron content of biofortified beans, without a change in native phytic acid content, did not result in higher iron absorption (8), and the iron fortification of foods has also shown little or no increase in iron absorption (10–12).

In selection and plant breeding programs, the existence of natural variations for any specific trait is a prerequisite. Since India is a secondary center for the diversity of finger millet, this study included a diverse core collection (33) that consisted of 623 accessions developed from 5940 accessions of finger millet from 23 countries, representing geographical regions and biological races from the entire collection. This core collection was extensively phenotyped in the first production season and the stability of GPA was evaluated using a smaller, but representative set of 275 accessions in the second production season. Finally, through a careful analysis of the iron and GPA content of a further selected 19 accessions, an accession that had a low GPA content was selected, along with a high GPA control, and further evaluated for their ability to breed true in a third production season. This approach also had the benefit of circumventing the deep concerns, including the social ramifications of farmer autonomy around crop genetic modification strategies.

Genetic modification strategies for reducing the GPA content operate through the identification of induced mutations in conventional varieties, or through the genetic engineering of enzymatic genes or transporters involved in phytic acid biosynthesis and storage, to yield *low phytic acid* (*lpa*) mutants. In several crops, *lpa* mutants have been identified, including maize, rice, wheat, barley, common bean, and soybean (53) by induced mutagenesis. Any reduction in GPA content is expected to have a negative impact on important plant phenological developments such as reduced germination, seedling survival, and increased susceptibility to stresses leading to reduced seed yield (53). Transgenic approaches have also been tried, for example, embryo-specific silencing of the multidrug resistance associated protein ABC transporter gene resulted in reductions in phytic acid content by 68–87% in maize and 37–90% in soybean (54). The much larger reduction of GPA content in transgenic lines renders them as an ideal material to examine the negative effects of low GPA content. But, this may perhaps be delayed or not permitted under the present restrictions on cultivation or consumption of genetically modified food grains in most countries, especially in India.

The selected low GPA accession in the present study did not have reduced germination and seedling establishment and yielded only 10% less than the most widely cultivated finger millet variety in the region (GPU 28). These observations suggest that a marginal 30% reduction in GPA content may not alter seedling vigor. From a farmers' sustainability perspective, the selection of accessions that naturally accumulate a low content of phytic acid, with a small yield penalty, seems a promising strategy. Since the variation in GPA values was naturally encountered, these contrasts formed an excellent set of accessions to examine iron absorption through clinical trials using human subjects. The two selected accessions were distinct even in their genomic sequences evidenced through SNP analysis using the available whole genome sequence information. Though there are 18 well-characterized genes involved in biosynthesis and transport/storage of phytic acid, Myo-inositol PO_4_ synthase (*MIPS*), that catalyzes the first step in phytic acid biosynthesis, that is, Glu 6-PO_4_ to Myo-inositol PO_4_, and the gene coding for the ABCC transporter that transports phytic acid to the protein storage vesicles (PSV) has been suggested as rate limiting steps (26). Hence, sequence variations in MIPS and ABC transporter genes were analyzed. Interestingly, there were no sequence variations in the MIPS gene among the 19 accessions and we found one nonsynonymous SNP in the ABCC transporter gene at the 1408 position. This variation resulted in an amino acid change from aspartic acid to histidine in the transporter domain that could affect the function of *EcABCC* phytate transporter protein. In support of this, changes in amino acid from glutamic acid to lysine, which represents a similar group change of amino acids, in the transmembrane domain of *Pvmrp1* (Phaseolus) (55) were associated with reduced phytic acid accumulation in protein storage vacuoles in which phytic acid is also stored.

Even though the iron absorption increased almost 3-fold from the low GPA content accession, the absolute values observed in the present study were relatively low. It is important to note that the present study subjects had a normal iron status and Hb, where iron absorption will be down regulated by the adequate body iron status. In women with ID, an earlier iron absorption study from a market-variety finger millet with a GPA content of 650 mg/100 g, eaten in the form of a steamed ball, showed that the iron absorption was higher, at 4.6% (32). An additional reason for the higher iron absorption in the earlier study could have been the study design with a single meal with a higher isotopic iron dose, which could result in higher isotopic iron absorption (32). The present study design was careful to avoid this by using three meals, such that the added isotopic iron was much lower, resembling the phytate: iron molar ratios present in a habitually consumed meal. The iron absorption findings in the normal subjects of this study can be extrapolated to a population with ID where the absorption is likely to be upregulated. Serum ferritin concentrations have a close inverse relation with iron absorption (56), such that observed iron absorption values can be corrected to a value of serum ferritin concentration present in a population with a high IDA prevalence. In a large survey of women in the Indian state of Uttar Pradesh (57), the geometric mean of inflammation adjusted serum ferritin concentrations was 15.9 μg L^−1^. When corrected (56, 58) to a ferritin value of 15 μg L^−1^, the iron absorption for the two accessions in this study was 6 vs. 2% (low GPA vs. high GPA) and will be higher as the iron status declines.

Finger millet was chosen for this study as it is a drought hardy crop species and leaves a lower water footprint on diets; indeed, finger millet is even seen as a “superfood” and a nutraceutical food. From an iron transaction view, the low GPA accession (with an iron content of 3.6 mg 100 g^−1^) when consumed in two meals of 100 g each, could contribute 0.3 mg iron daily to the physiological requirement. In contrast, the high GPA variety would provide only a third of this amount of iron. Improving diet diversity to increase the intake of absorption enhancers such as vitamin C, or behavioral modifications such as limiting the intake of inhibitory polyphenol rich beverages like tea with a meal (22, 59) are useful complementary strategies. From a food system perspective, the modest increase in iron absorption with a reduction in GPA content aligns with the principle that public health nutrition initiatives should necessarily be restrained to avoid the risks of over-nutrition or other unseen risks. Introducing such seeds into the seed market takes time, but once done, cannot be easily reversed.

Yield is a major concern of farmers, and while the yield of the low GPA accession was slightly (10%) lower than the market variety of finger millet, a village-level evaluation of the acceptability of a theoretically higher priced, but more iron-effective finger millet in Karnataka, India, showed that people were willing to pay more for millet that they felt had a health benefit (60). It is likely that with effective outreach and extension, a seed with a higher health benefit might fetch a higher price, offsetting the slightly lower yield. Further evidence of benefit from the selected low GPA content accession, through longer term feeding trials, is also required.

## Conclusion

This study leveraged the natural variation in GPA content in among the germplasm to identify a low GPA finger millet accession with improved iron absorption, without genetic modification. This accession can either be developed into a cultivar or used as a trait-donor genotype in focused breeding programs to further improve popular varieties of finger millet.

## Data Availability Statement

The datasets presented in this study can be found in online repositories. This data can be found at the “DDBJ“ data base here: https://ddbj.nig.ac.jp/resource/bioproject/PRJDB8131 and at: https://ddbj.nig.ac.jp/resource/bioproject/PRJDB10731.

## Ethics Statement

The studies involving human participants were reviewed and approved by the Institutional Ethical Committee at St. John's Medical College, Bengaluru, India (IERB No.: 87/2015). The patients/participants provided their written informed consent to participate in this study.

## Author Contributions

BR, SS, AK, DM, and MZ conceptualized the entire research. BR, NH, YN, RR, MT, SP, GK, and SS were involved in screening, cultivation, GPA analysis, and final selection of the low GPA accession. BR, MH, LP, KS, RS, and SS were involved in sequencing and *in silico* protein structure analysis. PT, BR, BB, and AK were involved in the human testing of iron absorption. All authors contributed to the preparation and editing of the manuscript.

## Funding

This research was supported by Indo-Swiss collaboration in Biotechnology Program (BT/IC-2/ISCB/Phase-IV/03/RAGI/2014; Department of Biotechnology, Government of India and the Swiss Agency for Development and Cooperation, Switzerland), and Swiss National Science Foundation Sinergia project (Number: 183578). BR acknowledges the Science and Engineering Research Board (SERB), Department of Science and Technology, Government of India for awarding the National Postdoctoral Fellowship. File No. PDF/2016/000639.

## Conflict of Interest

The authors declare that the research was conducted in the absence of any commercial or financial relationships that could be construed as a potential conflict of interest.

## Publisher's Note

All claims expressed in this article are solely those of the authors and do not necessarily represent those of their affiliated organizations, or those of the publisher, the editors and the reviewers. Any product that may be evaluated in this article, or claim that may be made by its manufacturer, is not guaranteed or endorsed by the publisher.
