# Supplementary material for: A Natural Low Phytic Acid Finger Millet Accession Significantly Improves Iron Bioavailability in Indian Women
Source: Front Nutr. 2022 Mar 24;8:791392. doi: 10.3389/fnut.2021.791392 (PMC8988890; doi:10.3389/fnut.2021.791392)
Supplement: Supplementary file 2 [file Data_Sheet_2.docx]

**Supplementary Text**

**A. crop production activities and the measurement of phenotypic traits**

The 623 germplasm accessions were sown in an augmented design consisting of 32 blocks with three checks in each block. Each block consisted of 23 rows with 2 m row length. Spacing between rows was maintained at 0.3 m and was 0.1 m between plants. Recommended doses of fertilizer (50 kg N ha^-1^, 40 kg P ha^-1^and 25 kg K ha^-1^) were applied using straight fertilizers (urea, single super phosphate and muriate of potash, respectively for nitrogen, phosphorus and potassium). Nitrogen was applied in split doses with half of recommendation applied at the time of sowing and the remaining half 45 days after sowing. Other intercultural operations like weeding and irrigation were followed to raise a healthy crop.

Phenotyping for 18 quantitative traits was carried out and trait values were recorded following guidelines defined by International Board for Plant Genetic Resources (IBPGR, 1985). Growth parameters such as plant height (cm) and specific leaf weight (ratio of dry weight of leaf to known leaf area expressed as mg cm^-2^) were recorded at the 50 % flowering stage. Day of ear head emergence (days) was recorded when 50% of plants in each accession showed complete emergence of the panicle. At harvest, tillers bearing ear-head were counted as productive tiller number and the ratio of productive tillers to total tiller number was considered as productive tiller ratio. Leaf number (average number of leaves plant^-1^), leaf dry weight (average oven dry leaf weight, g plant^-1^), straw dry weight (average oven dry weight of shoot biomass, g plant^-1^), and yield traits such as ear head weight (average weight of all the ear heads, g plant^-1^), mean ear head weight (average weight of ear head on main tiller, g ear head^-1^) and single plant yield (average weight of grains, g plant^-1^) were also recorded at harvest. The data for the above-recorded traits are represented as an average of that observed from 5 plants. Post-harvest, test weight (1000 seed weight, g) and threshing percentage (ratio of the grain yield to the ear head weight and expressed as per cent) were recorded. Parameters like the total leaf area obtained as the product of specific leaf Area and leaf dry weight; leaf area index as the ratio of leaf area to ground area; total dry matter as the sum of the dry weights of leaf, straw and grain yield (g) and harvest index as the ratio of grain yield to the total dry matter were also calculated.

**B. Molecular diversity analysis of the 623 core accessions**

Previously reported 35 Simple sequence repeat (SSR) markers from finger millet with amplicon size ranging between 150 to 500 base pairs (bp) were used to assess the molecular diversity of the 623 core accessions. The genomic DNA was isolated from young leaves, which were stored at -80˚C after collection from 30-day old seedlings, using the Cetyl Trimethyl Ammonium Bromide (CTAB) method described by Dellaporta et al., 1983. The Polymerase Chain Reaction (PCR) was performed in an Eppendorf nexus gradient (Eppendorf, Hamburg, Germany) using a 10 μl reaction mix containing 1.0 μl of 50 ng of genomic DNA template, 1.0 μl of 10X Taq buffer (with 25 mM MgCl_2_), 1.0 μl of 2 mM dNTPs, 0.3 μl of 3 units μl^-1^ Taq DNA polymerase (MBI Fermentas, USA) 0.5 μl of 10 pmole each of forward and reverse primers and 5.7 μl of sterile water. The temperature cycle of the PCR had an initial cycle of denaturation at 95˚C for 5 min, followed by 35 cycles of denaturation at 95˚C for 30s, annealing at 46 - 60˚C (for 35 different primers) for 45s, extension at 72˚C for 1 min, and a final cycle of extension at 72˚C for 8 min. Amplicons were resolved on 1.5% agarose gel stained with 0.5 μg.mL^-1^ ethidium bromide (Sigma Aldrich, St. Louis, MO USA) and visualized using a gel documentation unit Alpha imager (Cell Biosciences Inc., Santa Clara, CA). Scoring was performed based on presence or absence of amplicon of each allele in all the accessions.

**C. Next generation optical genome mapping for improving the reference genome sequence of PR 202.**

The Bionano Prep^TM^ Plant Tissue DNA Isolation Kit was used following the Bionano Prep^TM^ Plant Tissue DNA Isolation Base Protocol (Document number: 30068, Document revision: D, Bionano Genomics, Inc., San Diego, CA). Shortly, 1 gram of young leaves at two months after germination stage were fixed in formaldehyde to protect the nuclei and DNA against mechanical shearing. The leaves were then homogenised with a rotor-stator to break the tough plant cell wall and was followed by a density gradient purification for intact nuclei recovery. These were embedded in low melt agarose plugs for proteinase K (QIAGEN AG, Hombrechtikon, Switzerland) and RNAseA (QIAGEN AG, Hombrechtikon, Switzerland) digestion and subsequent purification of high molecular weight (HMW) DNA. The resulting HMW DNA was split into two staining protocols.

For the first, the Bionano Prep Direct Label and Stain (DLS) kit was used following the Bionano Prep Direct Label and Stain (DLS) Protocol (Document number:30206, Document revision: E, Bionano Genomics, Inc., San Diego, CA). Briefly, 750ng of HMW DNA was labeled using DLE-1 enzyme, followed by a Proteinase K digestion (QIAGEN AG, Hombrechtikon, Switzerland), clean-up and backbone labelling step. Instead, the second labelling entailed a customised protocol developed by Dr. Yuval Ebenstein in Tel Aviv. Summarily, 300 ng of HMW DNA was nicked using Nt.BspQI (New England Biolabs, Ipswich, MA, USA) and labeled using the Bionano-NLRS labelling Kit (BioNano Genomics, Inc. San Diego, CA). The labeled nicks were repaired to restore strand integrity. To generate methylation sensitive labeling profiles additionally to the NLRS-labelling, M.TaqI was added as it is a methyltransferase enzyme with a recognition site, TCGA. M.TaqI methylates the adenine residue within this sequence, and can be used to incorporate a labeled cofactor (Grunwald, et al. 2015). Thus, DNA was incubated with cofactor AdoYnCF640R and M.TaqI enzyme for five hours at 65°C and Proteinase K (QIAGEN AG, Hombrechtikon, Switzerland) was added for another two hour incubation at 45°C. The double-labeled DNA was then re-embedded in low melt agarose plugs for several washing steps using the Bionanogenomics Wash Buffer to remove any excess reagents. These plugs were digested and the DNA backbone was labelled using the Bionano-NLRS labelling Kit (BioNano Genomics, Inc. San Diego, CA) for backbone visualization. The resulting molecules were stained green for the Nt.BspQI restriction sites and red for methylated cytosines by M.TaqI with a blue backbone.

Labeled nicks were subsequently detected as dots on a string when run through the nanochannels of a Saphyr Chip G1.2 on the Bionano Saphyr System Instrument. These labelling reactions provided sequence specificity for an optimal label density of 10-13 labels per 100kbp. For this publication only the labeled DLE-1 and Nt.BspQ1 sites were used for subsequent analysis. The DLE-1 and nicked-labeled-HMW-DNA were run each on a Bionano Saphyr flowcell. DLE-1 data resulted in 860.36 Gbp of output and molecules with an overall N50 of 0.1624 Mbp. The yield for Nt.BspQ1 was instead 444.95 Gbp with an N50 0.0825 Mbp.

Based on the previously published genome reference (Hatakeyama, et al., 2018),

we assembled the first optical genome map with DLE1 enzyme by using Bionano Solve (ver. 3.3) and assembled the second optical genome map with Nt.BspQI enzyme on Bionano access server. Subsequently, these two maps were used for the hybrid scaffolding with the previous assembly (Hatakeyama, et al., 2018) by Bionano Solve (ver. 3.3) with default parameters.

**Homeolog gene annotation and sub genome classification**

Genes were predicted and annotated by Maker2 pipeline (ver. 2.9.31) (Cantarel, et al. 2008). Next, we aligned *E. indica* DNA reads (Illumina paired-end with 200 base insertion) to the newly assembled genome reference after trimming and filtering adapter sequences and low quality reads by using Trimmomatic (ver. 0.36) (Bolger, et al. 2014). Secondly, the coverage depth only in the gene regions were calculated. Combined the coverage data with the homeolog candidates that were searched by reciprocal BLAST best hits found in two different scaffolds (NCBI BLAST ver. 2.8.1+), the one which had higher coverage depth than another gene in each homeolog pair was assigned as a gene derived from *E. indica* as A-subgenome, and the other was assigned as a gene from another diploid progenitor as B-subgenome. The assembled genome size became 1,231,411,586 bases and the N50 length was 23,885,297. The total number of annotated genes was 62,554 (31,664 genes and 29,978 genes in A-subgenome and B-subgenome, respectively, and 912 genes were assigned neither A- nor B-subgenome).

**D. SNP validation by *In silico* analysis and protein structure prediction**

To validate the SNPs further, gene coding regions were predicted from each genome sequence of GE 2358 and GE 1004 and sequence specific primers were designed to encompass the SNP regions. The PCR amplified fragments were sequenced by Sanger’s method. Amino acid sequences were deduced for variant alleles from the two accessions (<https://www.ncbi.nlm.nih.gov/orffinder/)>. The amino acid sequences were subjected to BLASTp analysis to assess the homology with different crop species. ClustralW (<https://www.ebi.ac.uk/Tools/msa/clustalo/)> was performed for comparing both nucleotide and amino acid sequences for variant alleles. Functional domains in the proteins were identified by using hmmscan (Ver. 3.3.1) (<https://www.ebi.ac.uk/Tools/hmmer/search/hmmscan>). *In silico* structural analysis of the variant alleles of the two accessions were performed by predicting 3-dimensional structures of amino acid sequences using the robetta protein structure prediction server (<http://robetta.bakerlab.org>) and 3D structures of the predicted model were visualized using EzMol, a web based molecular visualization tool (<http://www.sbg.bio.ic.ac.uk/~ezmol/)>.

**E. Test meal recipe and its preparation for human feeding trails**

A wet dough was prepared by mixing finger millet flour (80 g) with deionised water (50 mL) and other ingredient such as onion (40 g), green chilli (2 g), coriander leaves (4 g), and salt (1.5 g), then equally divided into two parts and used for preparation of two flat breads. They were cooked with 6 g of oil, in a teflon coated frying pan for 10 min. The cooked *Ragi roti* was cooled to room temperature, weighed and wrapped in aluminium foil and stored at -20^°^C until the day of consumption. On the study day, the test meal was thawed for 3 hours at 4^⁰^C and warmed in a microwave oven for 30 seconds and 5 g of butter was added on top of the *Ragi roti* before administering it to the subjects.

**F. Measurements of Hb, serum ferritin and CRP in whole blood samples**

The Hb concentration in whole blood was measured immediately after blood collection by non-cyanide spectrophotometric method using an automated hematology analyzer (ABX Pentra 60 C+, Horiba Diagnostics, France). Bio-Rad trilevel hematology controls were used for quality control with an inter- and intra-day precision of 1.3%, 1.4%, 1.1% and 0.8%, 0.9%, and 0.9% for level 1, 2, and 3 control material respectively. Serum ferritin was analysed using an electrochemiluminescence immunoassay (E411, Roche Diagnostics, USA). Bio-Rad immunoassay plus trilevel controls were used for quality control, with an inter and intra-day precision of 1.6%, 1.3%, 1.1% and 2.2%, 1.1%, and 1.4% for level 1, 2 and 3 control material respectively. Serum CRP was measured by immunoturbidimetry (Cobas Integra 400 plus, Roche Diagnostics, USA). Two level instrument-specific reference materials were used for quality control. The inter- and intra-day precision were 2.1%, 2.5% and 1.1%, 2.8% for level 1 and 2 respectively

**G. Blood sample preparation for iron isotope analysis using NTIMS**

The mineralization of an aliquot of the thawed blood sample (0.75 mL) was performed by microwave digestion using 10 mL conc. HNO_3_ (69-70% JT Baker, USA) and 2 mL H_2_O_2_ (30%, EMPLURA, Merck, India). After mineralization, the solution was dried and dissolved in 5M HCl for iron separation by ion-exchange chromatography using ion-exchange resin AG1-X8 (200–400 mesh, Biorad, USA). The mineral solution was transferred to columns containing the ion-exchange resin and rinsed with 10 mL 5M HCl. The iron in the sample was eluted with 10 mL of 1M HNO_3_ from the column. After evaporation of the eluted solution, the sample was re-constituted with 500 μL 6M HCl. The iron was extracted from this solution in three steps into di- ethyl ether using 1 mL of the solvent each time. The combined organic phases were finally evaporated to dryness and stored for mass spectrometric analysis. The iron isotopic composition of the samples was determined by NTIMS (Triton, Thermo, Bremen, Germany) with a multicollector system, using FeF^-4^ molecular ions and a rhenium double-filament ion source. The evaporation filament as well as the ionization filament were coated with BaF_2_ to promote the formation of negatively charged ions. The sample iron was loaded as FeF_3_ in hydrofluoric acid (40%) on top of the BaF_2_ layer on the evaporation filament and coated with a solution of AgNO_3_ in HF (20%). A commercially available Fe standard (IRMM 014) was used as the reference standard for the analysis; the external precision was 0.023% and 0.003%, and the internal precision SD values were 0.0000169 and 0.00000317 for ^57^Fe/^56^Fe and ^58^Fe/^56^Fe ratios respectively.

**References:**

1. Dellaporta SL, Wood J, Hicks JB. A plant DNA minipreparation: version II. 1983. *Plant. Mol. Boil.* *rep*. Sep 1;1(4):19-21.

2. Grunwald, A. et al. 2015. Bacteriophage strain typing by rapid single molecule analysis. *Nucleic acids Res.* **43**, e117 .

3. Hatakeyama, M. et al. 2018. Multiple hybrid de novo genome assembly of finger millet, an orphan allotetraploid crop. *DNA Res.* **25**, 39-47.

4. Cantarel, B. L. et al. MAKER:2008. an easy-to-use annotation pipeline designed for emerging model organism genomes. *Genome Res.***18**, 188-196.

5. Bolger, A. M., Lohse, M. & Usadel, B. 2014. Trimmomatic: a flexible trimmer for Illumina sequence data, *Bioinformatics* **30**, 2114–2120.
