# Supplementary material for: A Natural Low Phytic Acid Finger Millet Accession Significantly Improves Iron Bioavailability in Indian Women
Source: Front Nutr. 2022 Mar 24;8:791392. doi: 10.3389/fnut.2021.791392 (PMC8988890; doi:10.3389/fnut.2021.791392)
Supplement: Supplementary file 3 [file Table_1.DOCX]

**Supplementary Table 1.** List of SSR primers used for molecular diversity analysis in 623 core finger millet accessions.

|  | Primer name | Forward Sequence (5’-3’) | Reverse sequence (5’-3’) |
| --- | --- | --- | --- |
| 1 | fmestssr16 | gaggcatgcacgtacaacac | ggagggagggaattcacaat |
| 2 | fm77 | gggaggtcgactaagctg | aacttgtcctgcatcatctc |
| 3 | fmblestssr2 | tatgggacaatctgctcaaaga | agaaggcaggtgaacgtaactc |
| 4 | fmestssr17 | catctccatctccatctcca | aaggacgatcgcaaccag |
| 5 | fm-ssr-01 | gcgaaaacacaatgcaaaaa | gcgttggttggacctgac |
| 6 | ugep76 | gcacgtacggattcacattg | ggtacggagacatcgacacc |
| 7 | ugep77 | ttcgcgcgaaatataggc | ctcgtaagcacccacctttc |
| 8 | ugep26 | atggggttagggttccagtc | tgtccctcactcgtctcct |
| 9 | ugep21 | caattgatgtcattgggacaac | gtatccacctgcatgccaac |
| 10 | fmblestssr4 | ttgtctgggatgacttgaacag | agctcgtggtacttatggaagg |
| 11 | ugep24 | gccttttgattgttcaactcg | cgtgatccctctcctctctg |
| 12 | fmestssr29 | ccacctgctccatctacatct | aaggacgatcgcaaccag |
| 13 | fm70 | gagtctatgatccgctcttg | cacgaattagacatgggatt |
| 14 | fmblestssr32 | tcctttgccactttatccaatc | gaatggggtatgaatggaacac |
| 15 | fmestssr9 | aagctccaaatccacacgtc | gggactctagtttccgctttc |
| 16 | fmestssr14 | agatcggcagccactacatc | gagactgagaagccgtgcat |
| 17 | fmestssr10 | ggcggctgctagggttc | cgcctcaatcatgacaacac |
| 18 | ugep110 | aaattcgcatccttgctgac | tgacaagagcacaccgactc |
| 19 | fmestssr21 | cgctagtagtacatcacagcta | ctgatggcgtatgggagtct |
| 20 | fmestssr18 | catctccatctccatctcca | ggacttgaggcagttgcag |
| 21 | fmblestssr3 | ggatgaagtgagattccccata | caagctcttctgtgttgcactc |
| 22 | fmblestssr23 | agtcatctagcaggtgtcggag | agctaacgacccagctcctc |
| 23 | fmblestssr7 | ataatggaaacccttcaaccct | cttgcagaaaattgcacagaag |
| 24 | fmblestssr5 | agtgtatcaagagcaaggggaa | ccacatttacgaacaatctcca |
| 25 | fmblestssr20 | agaggaaccgctcattcagata | ggggtagaaagtctgggaaatc |
| 26 | fmblestssr38 | acgcccttcagatcatacctaa | aattacagaggcttggtcgaaa |
| 27 | fmblestssr43 | gaggcccacacacttcttagcga | ggagtatgggtagtggaaccagtg |
| 28 | fmblestssr19 | ccaacttctggttttaacgtcc | gtggtaacttttctggcacctc |
| 29 | fmblestssr41 | gactaacaggagaatgctcatgg | tgagtgaaggtaagcacttcctg |
| 30 | fmblestssr27 | tgcttccactgctacattgact | cagaccataccaaagatcacga |
| 31 | fmblestssr34 | aagaaacattgcactcatgtgg | catcagcgtttgtggtgtactt |
| 32 | fmblestssr8 | agaagccaatctccatcagaaa | aaataactgcactggggtgttc |
| 33 | fmblestssr22 | cgggacgttgacgctatttat | gctgtcaaatcagcaactaacg |
| 34 | fmblestssr35 | tgtggtcctgaaaccatgttag | acgtgaagaacaacagggtgat |
| 35 | fmblestssr30 | agttgggaggaaatccagacta | taccacgaactaaacacagcca |

**Supplementary Table 2.** Nutrient composition of the reference test meal for iron absorption measurement

| SI NO | Ingredients | Weight | Energy | Protein | Fat | Carbohydrate | Vitamin C | Polyphenols |
| --- | --- | --- | --- | --- | --- | --- | --- | --- |
|  |  | g | kcal | g | g | g | mg | mg |
| 1 | Ragi flour | 80 | 256.6 | 5.73 | 1.54 | 53.4 | 0 | 108 |
| 2 | Chopped onion | 40 | 22.7 | 0.73 | 0.24 | 4.6 | 2.7 | 14 |
| 3 | Chili | 2 | 0.9 | 0.05 | 0.01 | 0.13 | 1.9 | 1.1 |
| 4 | Oil | 6 | 54 | 0 | 6 | 0 | 0 | 0 |
| 5 | Coriander leaves | 4 | 1.2 | 0.14 | 0.03 | 0.08 | 1 | 2.4 |
| 6 | Salt | 5 | 0 | 0 | 0 | 0 | 0 | 0 |
| 7 | Butter | 1.5 | 36.5 | 0 | 4.1 | 0 | 0 | 0 |
|  | Total | 138.5 | 371.9 | 6.6 | 11.9 | 58.3 | 5.5 | 125.6 |

Source: Indian food composition tables^18^

**Supplementary Table 3.** Mean difference (MD %) Coincidence rate (CR%), variable rate (VR%) and Shannon–Weaver diversity index (H`)

of 19 traits in finger millet core collection (n=623) and diversity panel (n=350).

| SI No | Trait | MD (%) | VR (%) | CR (%) | H` | |
| --- | --- | --- | --- | --- | --- | --- |
|  |  |  |  |  | n=623 | n=350 |
| 1 | Ear head emergence (days) | 0.77 | 99.62 | 100.00 | 6.43 | 5.85 |
| 2 | Plant height (cm) | 1.14 | 107.46 | 100.00 | 6.42 | 5.84 |
| 3 | Productive tiller (no plant^-1^) | 2.83 | 105.65 | 100.00 | 6.39 | 5.81 |
| 4 | Unproductive tiller (no plant^-1^) | 6.73 | 106.10 | 100.00 | 6.10 | 5.51 |
| 5 | Productive tiller ratio (%) | 0.41 | 107.26 | 100.00 | 6.43 | 5.85 |
| 6 | Leaf number (no plant^-1^) | 4.51 | 106.42 | 99.57 | 6.38 | 5.79 |
| 7 | Leaf area (cm^2^ plant^-1^) | 6.86 | 108.84 | 99.80 | 6.24 | 5.66 |
| 8 | Specific leaf weight (mg cm^-2^) | 0.81 | 112.83 | 100.00 | 6.42 | 5.84 |
| 9 | Leaf dry weight (g plant^-1^) | 7.04 | 106.61 | 99.86 | 6.24 | 5.67 |
| 10 | Leaf area index | 6.81 | 108.94 | 99.80 | 6.24 | 5.66 |
| 11 | Stem dry weight (g plant^-1^) | 6.15 | 107.85 | 100.00 | 6.25 | 5.67 |
| 12 | Mean ear head weight (g ear^-1^) | 2.57 | 107.50 | 100.00 | 6.35 | 5.76 |
| 13 | Ear head weight (g plant^-1^) | 5.45 | 109.16 | 100.00 | 6.34 | 5.75 |
| 14 | Seed yield (g plant^-1^) | 5.06 | 109.69 | 100.00 | 6.34 | 5.74 |
| 15 | Total dry matter (g plant^-1^) | 6.32 | 108.66 | 100.00 | 6.25 | 5.68 |
| 16 | Threshing (%) | 0.72 | 106.14 | 97.09 | 6.43 | 5.85 |
| 17 | Test weight (g 1000 seeds^-1^) | 0.31 | 106.46 | 100.00 | 6.42 | 5.84 |
| 18 | Harvest Index | 1.36 | 111.39 | 100.00 | 6.29 | 5.72 |
| 19 | Grain phytic acid (mg 100g^-1^) | 1.38 | 100.21 | 90.52 | 6.43 | 5.85 |
|  | Mean ± SD | 3.54 ± 2.64 | 107.20 ± 3.16 | 99.30 ± 2.23 | 6.34 ± 0.09 | 5.75± 0.10 |

**Supplementary Table 4.** Comparison of mean, range and variances for 19 traits between the 350 finger millet diversity panel and subset of 275 successfully grown accessions.

|  | Trait | Range | | Mean | | | Variances | |
| --- | --- | --- | --- | --- | --- | --- | --- | --- |
|  |  | n=350 | n=275 | n=350 | n=275 | *p*-value* | n=350 | n=275 |
| 1 | Ear head emergence (days) | 45.0 - 79.0 | 45.0 - 78.0 | 60.3 | 60.2 | 0.80 | 40.2 | 35.0 |
| 2 | Plant height (cm) | 45.0 - 146.0 | 45.0 - 146.0 | 95.5 | 95.9 | 0.78 | 329.2 | 318.2 |
| 3 | Productive tiller (number.plant^-1^) | 1.4 - 8.4 | 1.4 - 8.4 | 4.0 | 4.1 | 0.69 | 1.6 | 1.6 |
| 4 | Unproductive tiller (number.plant^-1^) | 0.1 - 2.0 | 0.1 - 2.0 | 0.5 | 0.5 | 0.91 | 0.1 | 0.1 |
| 5 | Productive tiller ratio (%) | 60.0 - 100.0 | 60.0 - 100.0 | 90.0 | 90.2 | 0.82 | 55.3 | 55.3 |
| 6 | Leaf number (number.plant^-1^) | 12.2 - 96.0 | 12.2 - 96.0 | 38.4 | 38.8 | 0.69 | 194.5 | 185.6 |
| 7 | Leaf area (cm^2^.plant^-1^) | 277.1 - 4685.1 | 277.1 - 4685.1 | 1349.0 | 1370.8 | 0.69 | 406625.8 | 422012.4 |
| 8 | Specific leaf weight (mg.cm^-2^) | 0.5 - 10.0 | 0.5 - 8.8 | 6.0 | 6.0 | 0.85 | 1.3 | 1.2 |
| 9 | Leaf dry weight (g.plant^-1^) | 1.8 - 22.4 | 1.8 - 22.4 | 8.0 | 8.1 | 0.65 | 12.8 | 13.0 |
| 10 | Leaf area index | 0.9 - 15.6 | 0.9 - 15.6 | 4.5 | 4.6 | 0.67 | 4.5 | 4.7 |
| 11 | Stem dry weight (g.plant^-1^) | 6.9 - 104.7 | 6.9 - 104.7 | 34.2 | 35.1 | 0.47 | 223.7 | 239.7 |
| 12 | Mean ear head weight (g.ear^-1^) | 0.7 - 12.4 | 0.7 - 10.8 | 5.0 | 5.1 | 0.73 | 4.9 | 5.0 |
| 13 | Ear head weight (g. plant^-1^) | 2.9 - 53.9 | 2.9 - 53.9 | 19.7 | 20.2 | 0.57 | 87.5 | 89.1 |
| 14 | Seed yield (g. plant^-1^) | 2.0 - 43.4 | 2.0 - 43.4 | 14.4 | 14.7 | 0.58 | 51.2 | 52.9 |
| 15 | Total dry matter (g. plant^-1^) | 11.5 - 181.1 | 11.5 - 181.1 | 62.5 | 64.0 | 0.51 | 695.7 | 719.1 |
| 16 | Threshing (%) | 42.1 - 89.7 | 43.9 - 88.7 | 72.4 | 72.4 | 0.96 | 63.8 | 58.3 |
| 17 | Test weight (g. 1000 seeds^-1^) | 1.0 - 3.6 | 1.3 - 3.6 | 2.4 | 2.4 | 0.95 | 0.2 | 0.2 |
| 18 | Harvest Index | 0.05 - 0.45 | 0.05 - 0.45 | 0.2 | 0.2 | 0.85 | 0.005 | 0.005 |
| 19 | Grain phytic acid (mg. 100g^-1^) | 497.1 - 951.0 | 497.1 - 903.2 | 727.9 | 721.1 | 0.35 | 8269.2 | 8042.2 |

*Non-significant at *p*>0.05 based on ANOVA and post-hoc Newman-Keuls test

**Supplementary Table 5.** Validation of the GPA content of 19 accessions by measurements at ETH Zurich (2016) and UAS Bengaluru (2015 and 2016).

|  | Accessions | Grain phytic acid (mg 100 g^-1^) | | | | | |
| --- | --- | --- | --- | --- | --- | --- | --- |
|  |  | UAS 2015 | UAS 2016 | ETH 2016 | Mean | SD | CV (%) |
| 1 | GE 597 | 549.1 | 477.9 | 445.0 | 490.7 | 53.2 | 10.8 |
| 2 | GE 1640 | 624.8 | 559.8 | 546.0 | 576.9 | 42.1 | 7.3 |
| 3 | GE 2358 | 631.9 | 567.0 | 563.0 | 587.3 | 38.7 | 6.6 |
| 4 | GE 2624 | 740.1 | 576.6 | 582.1 | 632.9 | 92.8 | 14.7 |
| 5 | GE 1557 | 744.2 | 580.5 | 430.0 | 584.9 | 157.1 | 26.9 |
| 6 | GE 595 | 598.4 | 604.8 | 517.0 | 573.4 | 49.0 | 8.5 |
| 7 | GE 6336 | 603.9 | 626.9 | 517.1 | 582.6 | 57.9 | 9.9 |
| 8 | GE 2707 | 731.9 | 631.1 | 523.0 | 628.7 | 104.5 | 16.6 |
| 9 | GE 2002 | 708.7 | 635.3 | 594.0 | 646.0 | 58.1 | 9.0 |
| 10 | GE 3000 | 813.0 | 647.0 | 529.0 | 663.0 | 142.7 | 21.5 |
| 11 | GE 2447 | 712.9 | 647.9 | 587.0 | 649.3 | 63.0 | 9.7 |
| 12 | GE 2619 | 836.3 | 678.0 | 545.7 | 686.7 | 145.5 | 21.2 |
| 13 | GE 4448 | 797.4 | 682.5 | 574.3 | 684.7 | 111.6 | 16.3 |
| 14 | GE 2063 | 730.4 | 685.7 | 654.0 | 690.0 | 38.4 | 5.6 |
| 15 | GE 3630 | 723.4 | 702.4 | 626.2 | 684.0 | 51.1 | 7.5 |
| 16 | GE 3094 | 768.0 | 719.2 | 628.9 | 705.4 | 70.6 | 10.0 |
| 17 | GE 4172 | 872.5 | 784.1 | 776.0 | 810.9 | 53.5 | 6.6 |
| 18 | 1265-B | 746.8 | 784.8 | 661.0 | 730.9 | 63.4 | 8.7 |
| 19 | GE 1004 | 850.9 | 887.1 | 892.9 | 876.9 | 22.7 | 2.6 |

**Supplementary Table 6.** List of GPA biosynthetic pathway and transport genes from rice genome database (IRGPSV) and their corresponding matching sequences identified from whole genome of finger millet (PR 202).

| SI No | Rice gene id (IRGSP) | finger millet gene id |
| --- | --- | --- |
| 1 | Os02t0169900-01 | ga12586, ga23919, gb11869 |
| 2 | Os02t0169900-02 | ga12586, ga23919, gb11869 |
| 3 | Os02t0169900-03 | ga12586, ga23919, gb11869 |
| 4 | Os02t0466400-01 | ga00703, ga00705, gb08472, gb08474, gb19125, |
| 5 | Os02t0819400-01 | ga03316, ga16748, gb10146 |
| 6 | Os02t0819400-02 | ga03316, ga16748, gb10146, gb23416 |
| 7 | Os02t0819400-03 | ga03316, ga16748, gb10146 |
| 8 | Os03t0142800-01 | ga27098, gb15861, gb18557, gb20371, gb26274 |
| 9 | Os03t0142800-02 | ga19217, ga27098, gb14539, gb15861, gb18557 |
| 10 | Os03t0192700-01 | ga00058, ga26761, gb07816, gb18866 |
| 11 | Os03t0587000-01 | ga12586, gb06329, gb11869 |
| 12 | Os03t0726200-01 | ga08018, ga13294, ga26502, gb11120, gb19125 |
| 13 | Os03t0737701-01 | ga13385, gb11037 |
| 14 | Os04t0661200-01 | gb14061 |
| 15 | Os09t0572200-01 | ga03316, ga16748, gb10146, gb23416 |
| 16 | Os09t0572200-02 | ga03316, ga16748, gb10146, gb23416 |
| 17 | Os10t0369900-01 | ga00058, ga26761, gb07816, gb18866 |
| 18 | Os10t0576100-01 | ga00703, ga00705, ga07953, gb08472, gb08474 |

**Supplementary Table 7.** List of finger millet accessions in different clades from NJ tree and their GPA values for two production seasons.

|  | Accession | Grain phytic acid (mg 100 g^-1^) | | |
| --- | --- | --- | --- | --- |
|  |  | UAS 2015 | UAS 2016 | Mean GPA |
| Clade with low GPA accession | GE 2619 | 836.3 | 678.0 | 757.2 |
|  | GE 1640 | 624.8 | 559.8 | 592.3 |
|  | GE 2624 | 740.1 | 576.6 | 658.4 |
|  | GE 2358 | 631.9 | 567.0 | 599.5 |
|  | GE 597 | 549.1 | 477.9 | 513.5 |
|  |  |  | **Mean** | **624.2^a^** |
| Clade with High GPA accession | GE 1004 | 850.9 | 887.1 | 869.0 |
|  | GE 3094 | 768.0 | 719.2 | 743.6 |
|  | GE 2447 | 712.9 | 647.9 | 680.4 |
|  | GE 6336 | 603.9 | 626.9 | 615.4 |
|  | 1265-B | 746.8 | 784.8 | 765.8 |
|  | GE 3630 | 723.4 | 702.4 | 712.9 |
|  |  |  | **Mean** | **731.2^b^** |

Values in the parenthesis significantly different at *p<0.05* based *on student’s t-test*
