# Supplementary figures and images for: A Natural Low Phytic Acid Finger Millet Accession Significantly Improves Iron Bioavailability in Indian Women
Source: Front Nutr. 2022 Mar 24;8:791392. doi: 10.3389/fnut.2021.791392 (PMC8988890; doi:10.3389/fnut.2021.791392)

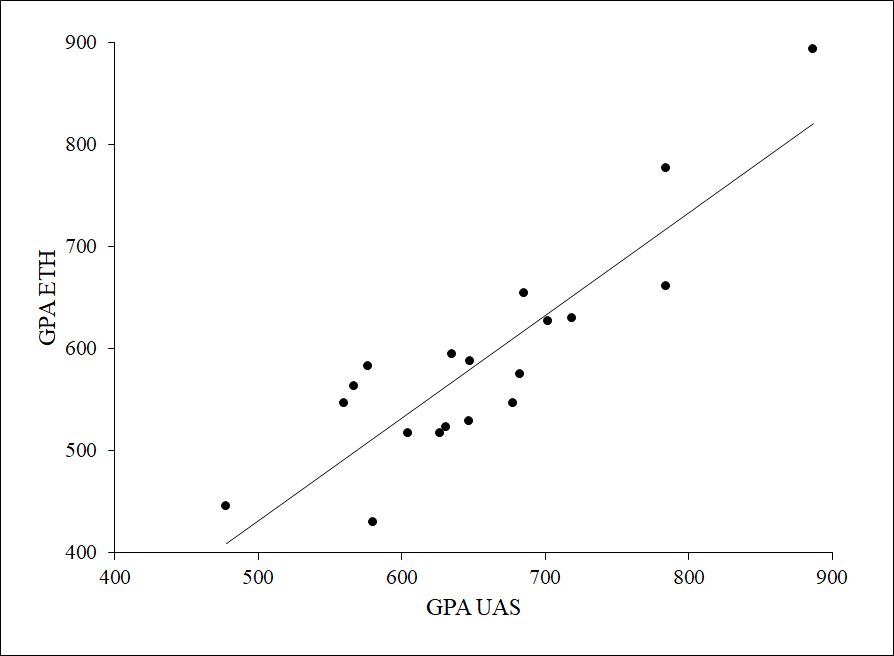

Supplement: Supplementary file 4 [file Image_1.TIF]

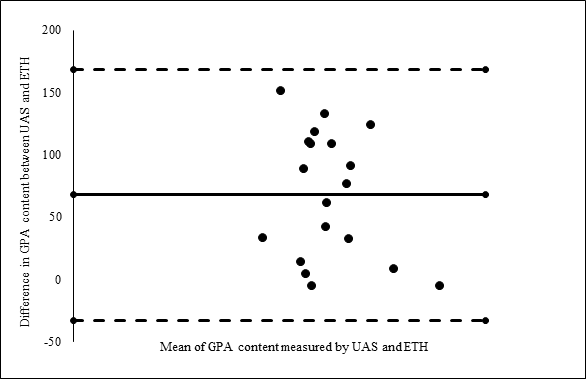

Supplement: Supplementary file 5 [file Image_2.TIF]

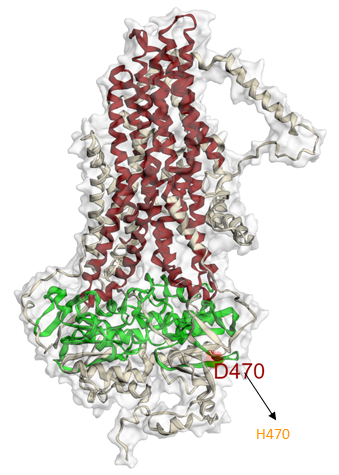

Supplement: Supplementary file 6 [file Image_3.TIF]
